# ^1^H-NMR spectroscopy identifies potential biomarkers in serum metabolomic signatures for early stage esophageal squamous cell carcinoma

**DOI:** 10.7717/peerj.8151

**Published:** 2019-11-29

**Authors:** Yan-Yan Liu, Zhong-Xian Yang, Li-Min Ma, Xu-Qing Wen, Huan-Lin Ji, Ke Li

**Affiliations:** 1 Department of Ultrasound, Shenzhen Bao’an Maternity & Child Healthcare Hospital, Shenzhen, Guangdong, China; 2 Department of Medical Imaging Center, the 2nd Affiliated Hospital, Shantou University Medical College, Shantou, Guangdong, China; 3 Department of Cardiothoracic Surgery, the 2nd Affiliated Hospital, Shantou University Medical College, Shantou, Guangdong, China; 4 Department of Public Health, Shantou University Medical College, Shantou, Guangdong, China

**Keywords:** Esophageal squamous cell carcinoma, Metabolomics, Biomarker, 1H- NMR spectroscopy

## Abstract

**Background:**

Esophageal squamous cell carcinoma (ESCC) is one of the most prevalent types of upper gastrointestinal malignancies. Here, we used ^1^H nuclear magnetic resonance spectroscopy (^1^H-NMR) to identify potential serum biomarkers in patients with early stage ESCC.

**Methods:**

Sixty-five serum samples from early stage ESCC patients (*n* = 25) and healthy controls (*n* = 40) were analysed using ^1^H-NMR spectroscopy. We distinguished between different metabolites through principal component analysis, partial least squares-discriminant analysis, and orthogonal partial least squares-discriminant analysis (OPLS-DA) using SIMCA-P+ version 14.0 software. Receiver operating characteristic (ROC) analysis was conducted to verify potential biomarkers.

**Results:**

Using OPLS-DA, 31 altered serum metabolites were successfully identified between the groups. Based on the area under the ROC curve (AUROC), and the biomarker panel with AUROC of 0.969, six serum metabolites (α-glucose, choline, glutamine, glutamate, valine, and dihydrothymine) were selected as potential biomarkers for early stage ESCC. Dihydrothymine particularly was selected as a new feasible biomarker associated with tumor occurrence.

**Conclusions:**

^1^H-NMR spectroscopy may be a useful tumour detection approach in identifying useful metabolic ESCC biomarkers for early diagnosis and in the exploration of the molecular pathogenesis of ESCC.

## Introduction

Esophageal squamous cell carcinoma (ESCC), a major histologic type of oesophageal cancer, is a prevalent upper gastrointestinal malignancy that affects major populations in China (Lin et al., 2013). Patients diagnosed during the early stages of ESCC have significantly greater long-term survival rates (at least five years or more) than those diagnosed at middle or later stages. Most ESCC patients exhibit metastasis or locally advanced ESCC at the time of diagnosis and have a five-year survival rate of only 5∼15% (Deng & Lin, 2018). Therefore, early detection of ESCC is important to improve survival rates and to predicate prognosis. Current techniques, including computed tomography scanning, upper gastrointestinal radiography, endoscopic ultrasonography, and chromoendoscopy with iodine staining, have limitations or low specificities and sensitivities (Kuwahara et al., 2010; Zhang et al., 2013a; Zhang et al., 2013b). These limitations highlight the need for accurate non-invasive screening tools to facilitate early ESCC detection. Thus, there is a need for the development of such a diagnostic tool, and for reliable biomarkers with high sensitivity and specificity at an early curative stage.

Metabolomics, a new high throughput technology, is a powerful approach for surveying endogenous small molecule metabolites (<1,000 Dalton) through the non-invasive analysis of cells, tissues, or biofluids (Idle & Gonzalez, 2007; Davis et al., 2012). Metabolomics focuses on the unique metabolomic fingerprint spectrum generated by metabolic processes in a biological system through targeted or non-targeted strategies (Sanchez-Espiridion et al., 2015; Xu et al., 2016). Metabolomics identifies a broad field for the detection of useful biomarkers for disease diagnosis, therapy, and prognosis, and for insights into the pathophysiologic mechanisms of oncogenesis and tumour staging. ^1^H nuclear magnetic resonance (^1^H-NMR) spectroscopy is a non-destructive and non-invasive technique that requires a small quantity of samples to screen early cancer-associated perturbations in cellular metabolism. Today, many studies are applying metabolomics technology on tissue, plasma, serum, and urine samples to reveal variation in the tricarboxylic acid (TCA) cycle, and in the metabolism of choline, amino acids, fatty acids, and urea to identify metabolite biomarkers in patients with oesophageal cancer (Yang et al., 2013; Mir et al., 2015; Cheng et al., 2017). However, there are still some questions that need to be answered. What are the critical metabolite changes that occur in the early stages of ESCC? What level of serum metabolomic sensitivity and specificity is required to distinguish patients with early ESCC from healthy groups? Answering these two questions may provide a means to improve early diagnosis, therapy, and prognoses for ESCC patients.

Therefore, we applied principal component analysis (PCA) and partial least squares discriminant analysis (PLS-DA) methods based on ^1^H-NMR spectroscopy to identify global changes in serum metabolic profiles. We analysed and compared the serum metabolic profiles between healthy controls (CTRL) and patients in the early stages of ESCC. Furthermore, orthogonal partial least squares discriminant analysis (OPLS-DA) was applied to visualize the metabolic variation between the two serum samples. Our objectives of this study were to identify potential diagnostic serum biomarkers for early stage ESCC using ^1^H-NMR spectroscopy, and to increase our understanding of the underlying mechanisms of ESCC.

## Materials & Methods

### Study subjects and sample collection

Serum samples (65) were collected from the Department of Cardiothoracic Surgery and the Medical Examination Center in the Second Affiliated Hospital of Shantou University Medical College, between December 2016 and June 2018. These samples included those from 40 CTRL and 25 patients in the early stages of ESCC (stage I/II) not treated with chemotherapy, radiotherapy, or chemoradiotherapy. Patient information and clinical characteristics are summarized in Table 1. The clinical stages of ESCC patients were diagnosed using esophagoscopy examination with biopsy, X-ray barium radiography, and chest computed tomography. Tumour staging was based on the American Joint Committee on Cancer’s (AJCC) 7th staging system. CTRL, recruited from our health examination center, were matched with patients with ESCC based on age, gender, BMI, and place of residence. This study was approved by the Ethical Committee of Second Affiliated Hospital of Shantou University Medical College (Registration No. 2016-32) and carried out in accordance with the Declaration of Helsinki. Informed consent was obtained from each subject before participation in the study.

**Table 1:** Summary of clinical and demographic characteristics for early stage of ESCC patients and healthy controls (CTRL).

	**CTRL**	**ESCC**	*p*
Number of subjects	40	25	0.502
Male/Female	31/9	19/6	–
Age (years)	61.6 ± 7.59	62.8 ± 8.36	0.721
BMI (kg/m^2^)	21.6 ± 5.03	22.3 ± 4.98	0.862
Differentiation degree	/	Well:23 Middle:2	–
Lymph node metastasis	/	Negative:19 Positive:6	–
TNM classification	/	I:7 II:18	–

**Notes.**

ESCC esophageal squamous cell carcinoma CTRL healthy controls

### Sample preparation and ^1^H NMR spectroscopy

About five mL of peripheral venous blood was collected from each subject between 7 and 8 am into tubes without any anticoagulant, and centrifuged at 3,000 rpm for 10 min. Serum samples were isolated and immediately stored in EP tubes for further analysis. Anticoagulation can reduce or inhibit the activity of protein and enzymes so we did not denature or filter them during NMR acquisition. Then, in order to avoid potential disturbance to the metabolic status during the denaturation or filtration processes, we acquired the serum sample and stored it at −80 °C immediately. In order to reduce the influence of the proteins in blood on the NMR acquisition, Carr-Purcell-Meboom-Gill (CPMG) pulse sequence was used in our study. This pulse sequence can suppress signals from macromolecules and other molecules with constrained molecular motion, although not completely. CPMG is also a common method to acquire NMR spectrum of blood samples and has aided in the metabolomic studies of many kinds of clinical disease (Zhang et al., 2013a; Zhang et al., 2013b; Chen et al., 2015). Frozen serum samples were thawed immediately and vortexed for 10 s at room temperature. Then, 200 µL of phosphate buffer solution (90 mM NaH_2_PO_4_/Na_2_HPO4, pH = 7.4) was added to 400 µL serum for NMR detection. After centrifugation at 10,000 rpm for 10 min at 4 °C, approximately 550 µL of the clear supernatant was transferred into five mm NMR tubes (ST500, NORELL, Inc., Morganton, NC, USA) for sampling. An NMR spectrometer (600.13 MHz, Bruker Avance III, Bruker Corporation, Kalsruhe, Germany) was used to obtain ^1^H-NMR spectra under CPMG pulse sequence with a total spin relaxation delay (2 n*τ*) of 70 ms to weaken broad resonances from high molecular weight compounds and to retain low molecular weight compounds and some lipids. All serum samples were analysed in random order at 298 K. One-dimensional spectrum was used to obtain CPMG spin echo pulse sequence (RD-90°-(*τ*-180°-*τ*)n-ACQ) to suppress water signal with a relaxation delay of 5 s. The acquisition parameters were: spectral width, SW = 20 ppm; recycle delay, RD = 4.0 s; t1 = 350 μs; mixing time, tm = 100 ms; number of scans, NS = 32; number of points, TD = 32,768; and acquisition time, AQ = 2.73 s.

### ^1^H NMR spectroscopy analysis

The raw data (free induction decays, FIDs) were input into MestReNova Version 9.0.1 (Mestrelab Research, Santiago de Compostela, Galicia, Spain, 9.0.1) for processing and complexity reduction to facilitate pattern recognition. To enhance the signal-to-noise ratio, all ^1^H-NMR spectra were multiplied by a 1.0 Hz exponential line broadening prior to Fourier transformation. The chemical shifts of serum spectra were referenced with the methyl doublet signal of lactate at *δ* 1.33 ppm as an internal standard. Both phase adjustment and baseline correction were performed manually. Each spectrum (from 9.0 to 0.5 ppm) was segmented into rectangular buckets with equal widths of 0.002 ppm with each excluding residual water (from 5.18 to 4.67 ppm) and urea (from 6.40 to 5.40 ppm) regions. To remove the dilution effect or bulk mass differences among samples due to differing serum weights, the remainder of each bucket was internally normalized to the total sum of the spectral integrals for each compound prior to pattern recognition analysis. In our study, one bin was only 1.2 Hz, therefore, no more than one compound existed in each bucket. In the usual case of more than one bucket per compound, each bucket was statistically analyzed in multivariate statistical analysis, but the sum of the integrals in any peak (not bucket) of some compound in NMR spectra was used for univariate statistical analysis.

### Pattern recognition (PR) analysis of serum

To establish a global profile of the feature in ESCC patients and CTRL, we used multivariate analysis to identify consistent variations between ^1^H NMR data sets. Serum spectra data were input into the SIMCA-P+ version 14.0 software package (Umetrics Inc., Umea, Sweden, V 14.0) for PR analysis. First, PCA, an unsupervised PR method, was performed using the Parato-scaled normalized ^1^H NMR spectra to discover the intrinsic trends and outliers between the two serum sample groups. Then, PLS-DA and OPLS-DA, the two supervised PR methods, were performed to prevent over-fitting of the statistical model and to select potential biomarkers.

Model quality and reliability were assessed using R^2^X, R^2^Y, and Q^2^ values, which reflect the explained variance and model predictability. R^2^X represents the variation explained by the models and R^2^Y indicates the ‘goodness of fit’ in the data. Q^2^, calculated by a cross-validation procedure, indicates the predictability of the model. To avoid model overfitting, a default seven-round cross-validation procedure was performed in SIMCA-P+ 14.0 to determine the optimal number of principal components. Reliability of the models was further rigorously validated by a permutation analysis (*n* = 300 times). The variable importance in the projection (VIP) from OPLS-DA models was identified as a coefficient for peak selection. These variables were considered potential biomarker candidates based on class discriminating information –the higher the value, the greater the discriminatory power of the metabolite. VIPs larger than 1.0 usually represent those metabolites with the greatest group discrimination.

### Data preprocessing statistical analysis

CV-ANOVA (analysis of variance testing of cross-validated predictive residuals) was performed to identify significantly different features between groups in OPLS-DA models. Univariate statistical significance of *p* <0.05 was considered to distinguish metabolites. Student’s *t* (normal distribution) or Mann–Whitney U (if abnormal distribution) tests were performed to analyse the metabolic profiles between ESCC patients and CTRL. The metabolites were recognized according to the Human Metabolome Database (http://www.hmdb.ca/) and receiver operating characteristic (ROC) analysis was conducted in SPSS 19.0 (SPSS Inc., Chicago, IL, United States) to verify potential biomarkers. Area under the ROC curve (AUROC) of >0.80 indicated excellent diagnostic ability.

## Results

### Metabonomic profiling of serum samples for ESCC patients and CTRL

**Figure 1:**
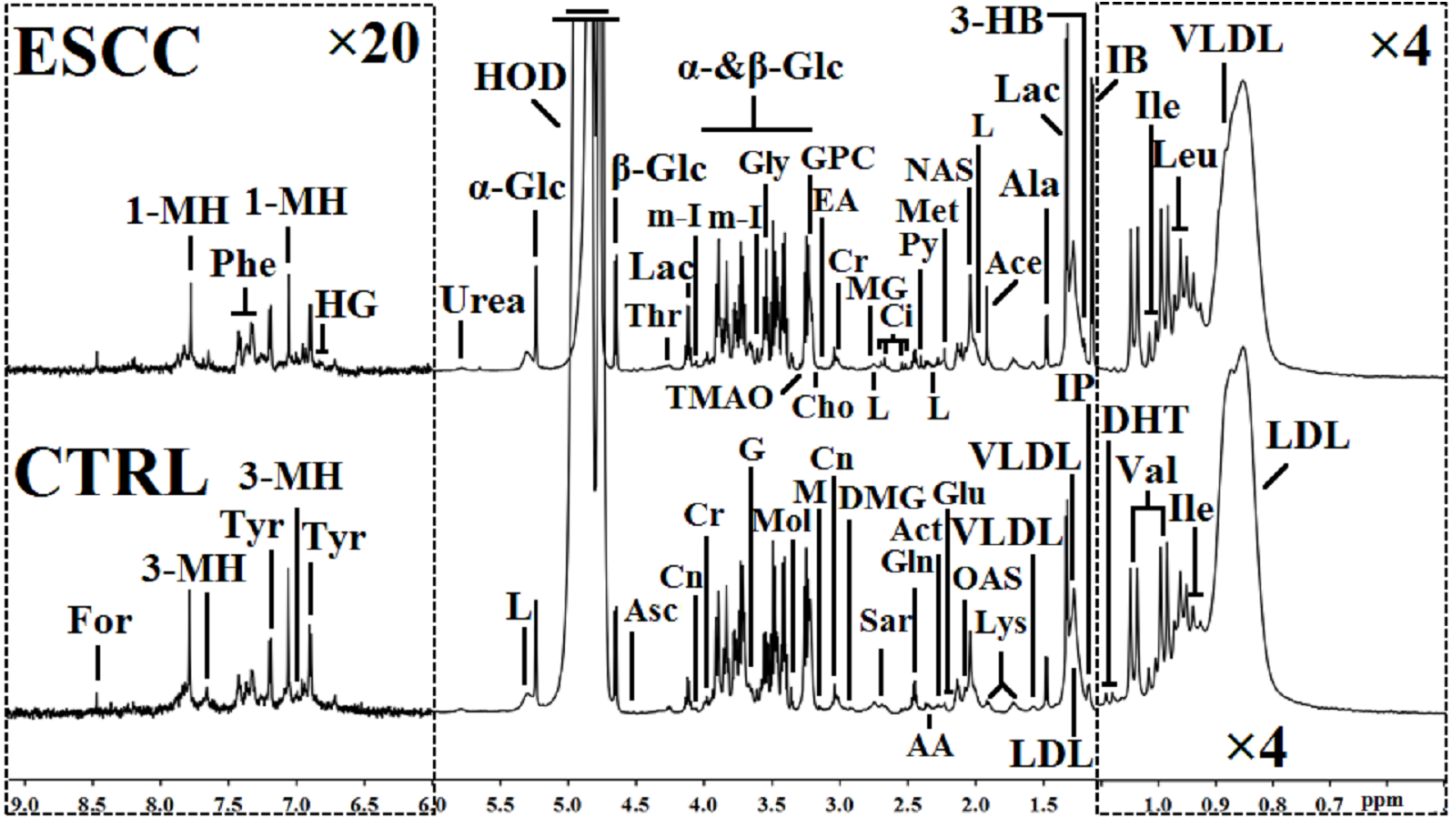
^1^H NMR spectra (*δ* 0.5–9.0 ppm) of serum obtained from healthy controls (CTRL) and the patients at the early stage of esophageal squamous cell carcinoma (ESCC). The regions of *δ* 6.0–9.0 ppm (in the left dashed box) were vertically magnified 20 times, and the regions of *δ* 0.5–1.1 ppm (in the right dashed box) were magnified four times both vertically and horizontally compared with corresponding regions of *δ* 1.1–6.0 ppm for the purpose of clarity. Keys: 1-MH, 1-methylhistidene; 3-HB, 3-hydroxybutyrate; 3-MH, 3-methylhistidene; AA, acetoacetate; Ace, acetate; Act, actone; Ala, alanine; Asc, ascorbate; Cho, choline; Ci, citrate; Cn, creatinine; Cr, creatine; DHT, dihydrothymine; DMG, N, N-dimethylglycine; EA, ethanolamine; For, formate; G, glycerol; Gln, glutamine; Glu, glutamate; Gly, glycine; GPC, glycerolphosphocholine; HG, homogentisate; HOD, the residual water resonance; IB: isobutyrate; Ile, isoleucine; IP, isopropanol; L, lipid; Lac, lactate; LDL, low density lipoprotein; Leu, leucine; Lys, lysine; M, malonate; Met, methionine; MG, methylguanidine; m-I, *myo*-inositol; Mol, methanol; NAS, N-acetyl glycoprotein signals; OAS, O-acetyl glycoprotein signals; Phe, phenylalanine; Py, pyruvate; Sar, sarcosine; Thr, threonine; TMAO, trimethylamine N-oxide; Tyr, tyrosine; Val, valine; VLDL, very low density lipoprotein; α-Glc, α-glucose; β-Glc, β-glucose.

The one-dimensional ^1^H-NMR spectra of serum samples provided an overview of all metabolites from ESCC patients and CTRL (Fig. 1). We did not try to absolutely quantify the metabolites in serum samples by comparing them with an internal standard such as DSS or TMSP. In fact, no current NMR standard is readily available for serum. DSS, TMS or TMSP are not suitable as an internal standard for proteinaceous fluids (such as serum and plasma) due to their potential protein binding capacity (Shimizu, Ikeguchi & Sugai, 1994; Alum et al., 2008). The chemical shift and line-width of DSS or TMSP will show large variation due to protein binding, thus greatly affecting the accurate qualification of the metabolites in serum. In our study, endogenous lactate (*δ* 1.33 ppm) in serum samples was used as an internal standard reference, and the relative concentrations of the metabolites were calculated and statistically discriminated by integrating their signals in the NMR spectra. Ultimately, approximately 47 metabolites were tagged in the spectra between the two groups, including amino acids, organic acids, energy metabolism, methylguanidine, myo-inositol, trimethylamine N-oxide, glucose components, lipids, carbohydrates, nucleotides and so on.

### PCA, PLS-DA, and OPLS-DA pattern recognition analysis of serum metabolomic profiling for ESCC patients and CTRL

**Figure 2:**
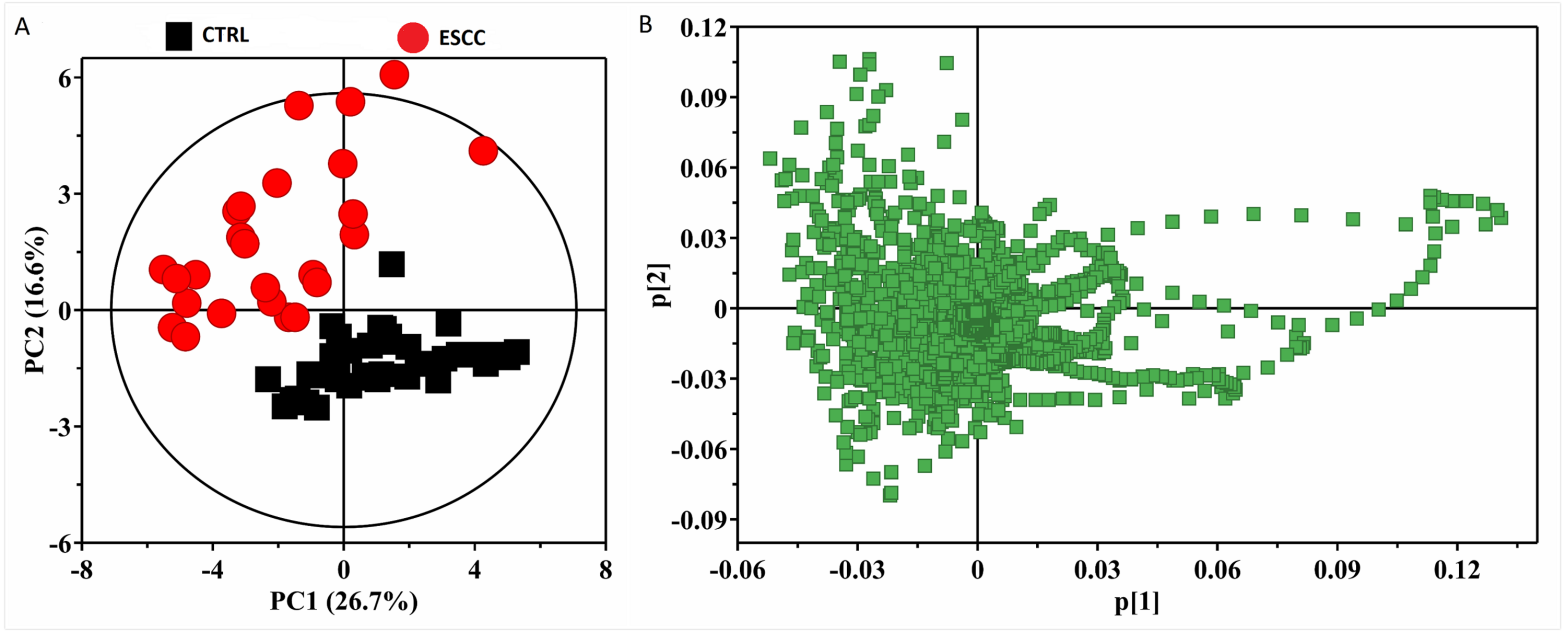
The 2D PCA scores plots (A) and loading plots (B) based on ^1^H CPMG NMR spectra of serum obtained from healthy controls (CTRL) and ESCC group (*R*^2^*X*(*PC*1 + *PC*2) = 43.3%).

**Figure 3:**
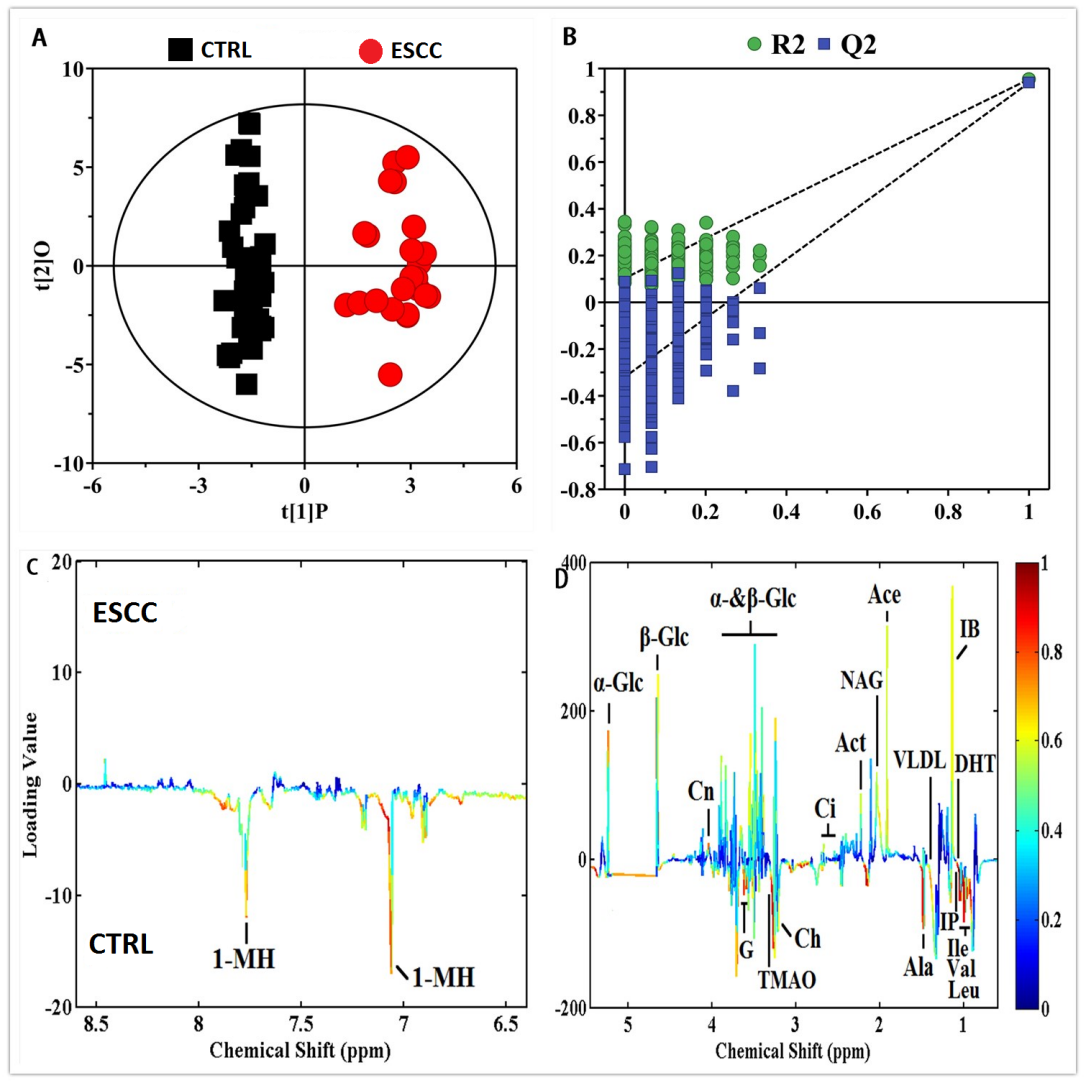
OPLS-DA scores plots (A) derived from ^1^H CPMG NMR spectra of serum and corresponding coefficient loading plots (C, D) obtained from controls (CTRL) and ESCC by cross validation (B) by permutation test. The color map shows the significance of metabolites variations between the two classes. Peaks in the positive direction indicate metabolites that are more abundant in the groups in the positive direction of first principal component. Consequently, metabolites that are more abundant in the groups in the negative direction of first primary component are presented as peaks in the negative direction.

To obtain useful metabolomic profiles, unsupervised PCA analysis of ^1^H-NMR results showed the difference between ESCC patients and CTRL. Pareto scaling, performed by dividing the mean-centered data by the square root of the standard deviation (SD), was applied to the variables. The two-dimension PCA score plots and loading plots revealed separation trends and group clustering based on ^1^H-NMR spectra of the two groups (R^2^X(PC1+PC2) = 43.3%) (Figs. 2A–2B). However, we were unable to identify a very clear difference between them via PCA scores plot. We performed supervised PLS-DA and the resultant plot showed different metabolic perturbations between the groups using 2D score plots (R^2^X = 51.9%, R^2^Y=80.9%, *Q*^2^ = 81.4%) (Fig. S1). To maximize the group separation and to visualize the metabolic distinctions, the OPLS-DA classification model was performed to investigate metabolomic alterations. We used the OPLS-DA methods to distinguish between ESCC patients and CTRL obviously (Fig. 3 A-D). Taken together, these results suggest that the models were robust and the random permutation tests (300 iterations) indicated that those models were not over-fitted (R^2^X = 81.9%, R^2^Y = 95.4%, *Q*^2^ = 94.0%, CV-ANOVA *p* = 2. 23 ×10^−35^). Our results indicate that ^1^H-NMR-based serum metabolomics have potential application in identifying early ESCC.

### Discovery, description, and identification of potential biomarkers and a biomarker panel

**Figure 4:**
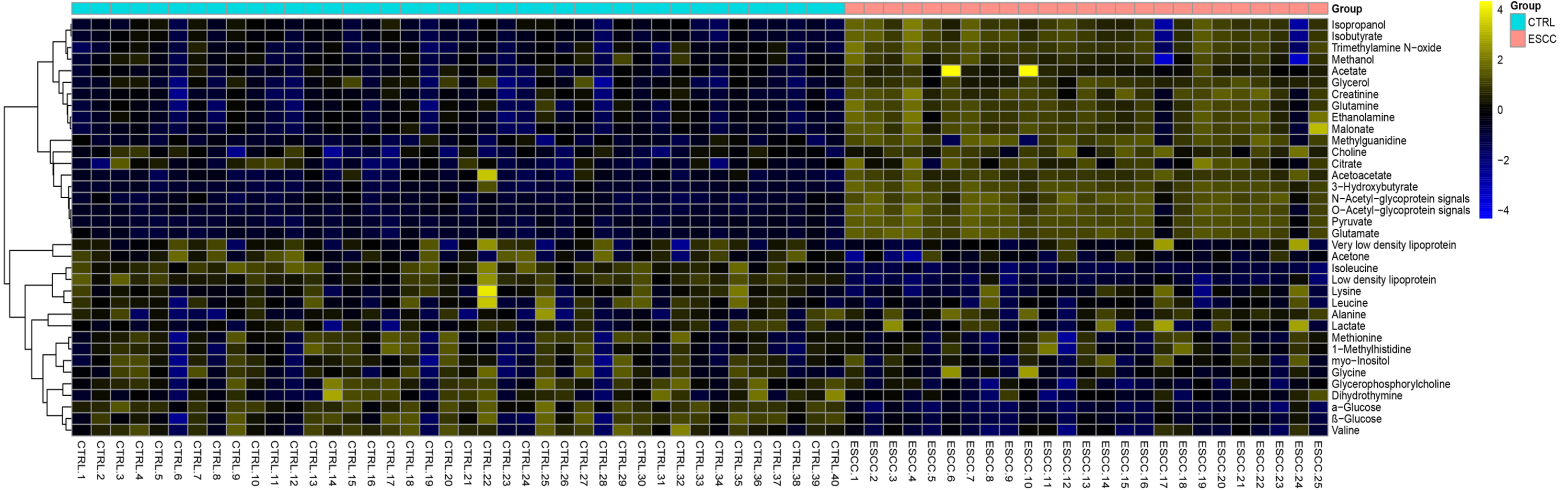
Hierarchical cluster analysis of serum metabolic profile for distinguishing ESCC from healthy controls (CTRL). The rows represent the results of the expression of metabolites, and the columns show serum samples. The expression values are represented by the color scale. The intensity increases from blue (relatively decreased) to yellow (relatively increased). In the top bar, the light blue color indicates CTRL, and the pink color indicates ESCC patients.

**Figure 5:**
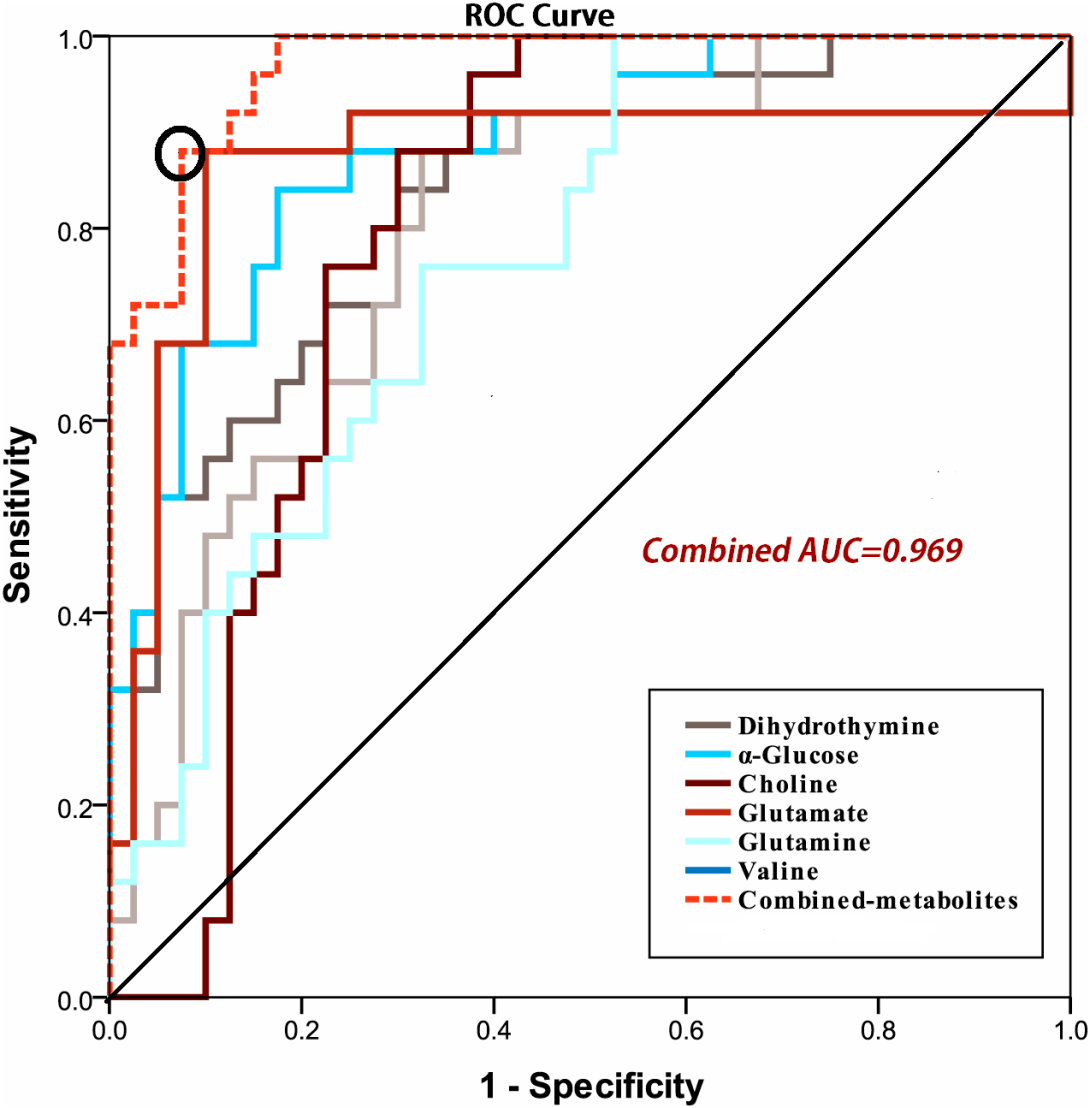
ROC curve of the discriminatory power of combined potential biomarkers panel for ESCC and CTRL (Combined AUC = 0.969).

**Figure 6:**
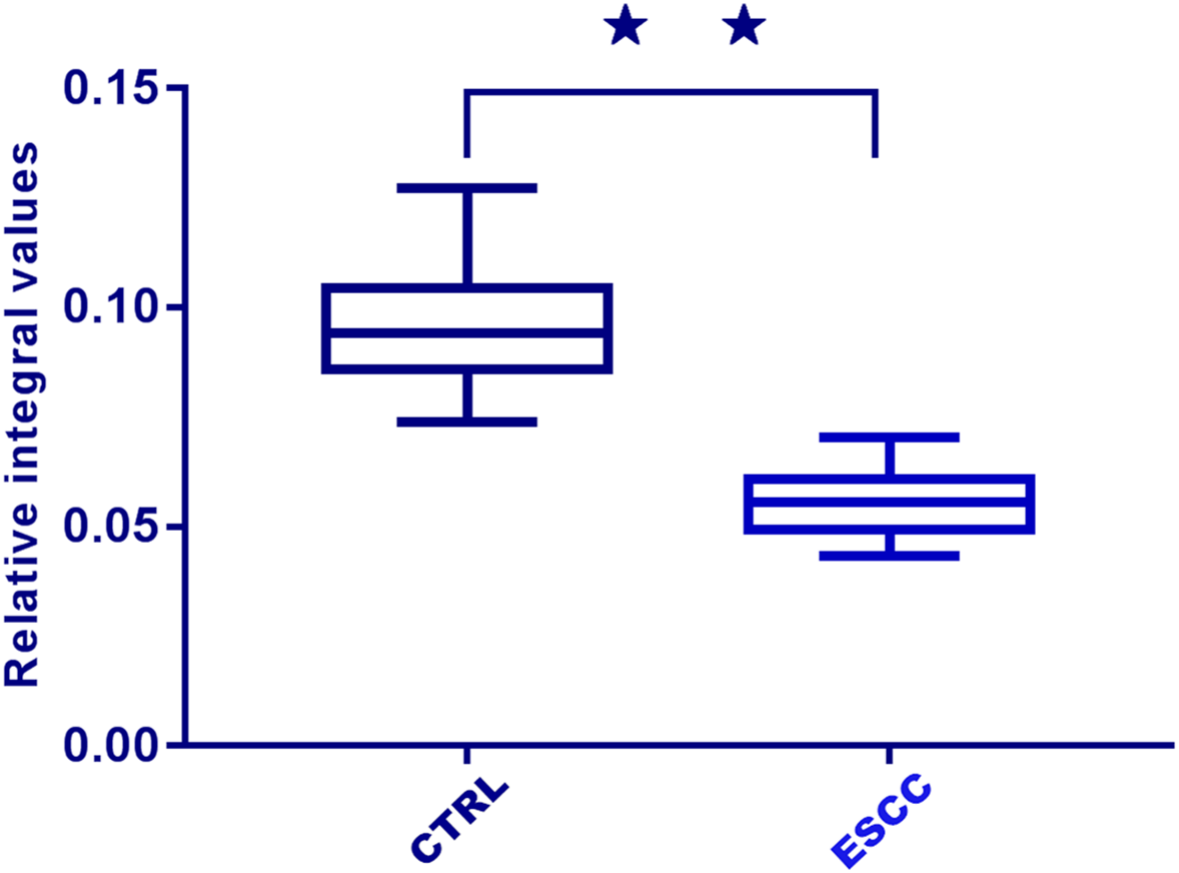
Box plots of relative integral values of dihydrothymine between healthy controls (CTRL) and ESCC groups (⋆⋆, *p* < 0.01).

A total of 31 differential metabolites were identified as characteristic metabolites (Table 2). We constructed a map of hierarchical cluster analysis (Fig. 4) to visualise the distinction power of biomarkers between the two groups. The rows represent the results of the expression of metabolites, and the columns show serum samples. In the top bar, the light blue color indicates CTRL individuals, and the pink color indicates ESCC patients. Figure 4 indicates that the metabolite profile could distinguish ESCC patients from CTRL. Based on the sensitivity and specificity of this approach, ROC analyses were performed for further prediction of potential biomarkers. Neoplastic diseases involve systematic disturbance of metabolic biochemical pathways. Therefore, a biomarker panel including multiple biomarkers, rather than a single biomarker, can better distinguish the different groups and supply useful information for clinicians. We identified a panel of six biomarkers, glucose, choline, glutamine, glutamate, valine, and dihydrothymine (DHT), that were combined together by binary logistic regression with high AUC values of 0.969 (Fig. 5). Changes in these metabolite biomarkers could be related to tumour burden. In particular, the decline of DHT (Fig. 6, *p* < 0.01), a new feasible biomarker, is associated with tumor invitation.

**Table 2:** Summary of metabolites statistical data from healthy controls (CTRL) and ESCC groups. The Variable importance in projection (VIP) values more than 1,000 were used for the statistical significance. Univariate statistical significance of *p* < 0.05 was identified to distinguish early ESCC metabolites from CTRL. Sensitivity, specificity, AUC curve value of the metabolites were also for discrimination ESCC from CTRL. Metabolites in bold showed potential biomarkers between ESCC and CTRL.

**Metabolites**	**ESCC vs CTRL**					
	**VIP**	***p***	**trend**	**sensitivity**	**specificity**	**AUC**
1-Methylhistidine	1.096	2.55 × 10^−8^	↓	0.694	0.783	0.754
3-Hydroxybutyrate	2.430	2.27 × 10^−13^	↑	0.712	0.754	0.712
Acetate	2.171	8.09 × 10^−6^	↑	0.649	0.525	0.637
Acetone	2.143	0.0005	↑	0.432	0.708	0.584
Alanine	2.516	0.0004	↓	0.753	0.822	0.789
Choline	3.819	2. 27 ×10^−21^	↑	**0.839**	**0.876**	**0.855**
Citrate	1.134	0.031	↑	0.587	0.682	0.633
Creatinine	1.023	2.66 × 10^−7^	↑	0.691	0.535	0.579
Dihydrothymine	3.055	3.25 × 10^−21^	↓	**0.845**	**0.822**	**0.824**
Glutamate	3.587	1.38 × 10^−10^	↑	**0.823**	**0.847**	**0.834**
Glutamine	3.135	1.43 × 10^−8^	↑	**0.803**	**0.841**	**0.816**
Glycerol	1.725	2.68 × 10^−5^	↑	0.662	0.713	0.678
Isobutyrate	1.543	1.54 × 10^−5^	↑	0.630	0.512	0.594
Isoleucine	1.891	6.73 × 10^−16^	↓	0.610	0.675	0.625
Isopropanol	1.938	0.0007	↓	0.723	0.641	0.662
Leucine	1.964	1.38 × 10^−11^	↓	0.749	0.810	0.786
Low density lipoprotein	2.061	0.017	↑	0.520	0.812	0.642
Lysine	2.406	7.83 × 10^−6^	↑	0.707	0.556	0.634
Malonate	1.191	2.48 × 10^−7^	↑	0.498	0.674	0.589
Methanol	2.273	1.36 × 10^−8^	↓	0.476	0.683	0.529
Methionine	1.378	1.96 × 10^−5^	↓	0.588	0.786	0.671
Methylguanidine	2.648	1.64 × 10^−10^	↓	0.801	0.721	0.742
myo-Inositol	1.573	0.003	↓	0.719	0.843	0.785/
Pyruvate	2.054	1.84 × 10^−9^	↓	0.723	0.810	0.773
Trimethylamine N-oxide	1.128	6.45 × 10^−6^	↓	0.675	0.497	0.576
Valine	2.964	6.45 × 10^−9^	↓	**0.801**	**0.843**	**0.827**
Very low density lipoprotein	2.234	0.014	↓	0.611	0.720	0.651
α-Glucose	4.672	3.05 × 10^−9^	↓	**0.891**	**0.856**	**0.879**
β-Glucose	3.656	0.0003	↓	0.853	0.769	0.798

**Notes.**

‘ ↑’, increased. ‘ ↓’, decreased.

## Discussion

Patients with ESCC have greater long-term survival when ESCC is treated in its early stages. Early detection increases diagnostic accuracy, promotes personalized treatment, and improves prognostic effects. However, the main causes of tumour occurrence remain unknown (Lee et al., 2017). One study found five salivary biomarkers (propionylcholine, N-Acetyl-L-phenylalanine, sphinganine, phytosphingosine, and S-carboxymethyl-L-cysteine) in combination with an AUC value of 0.997 differentiated between early stage oral squamous cell carcinoma and controls (Wang et al., 2014), while we found a panel of six serum biomarkers (glucose, choline, glutamine, glutamate, valine, and DHT) combined together with high AUC values of 0.969 differentiated between early stage ESCC and CTRL. The results showed that there were no obvious cross correlations between the two groups, indicating that different forms of cancer may have different metabolic features associated with them. Therefore, recognition of the characteristic metabolites of early stage ESCC would enable us to identify the disease and intervene earlier, which is more likely to prevent and/or delay the development of ESCC to medium-term or advanced stage, ultimately resulting in improved prognoses for patients. Previous studies found that variations in molecular and biochemical metabolism occur before histopathological and morphological changes (Wei, Wolin & Colditz, 2010). Here, we used ^1^H-NMR spectroscopy to identify useful metabolic ESCC biomarkers for early diagnosis, and to further explore the molecular pathogenesis of ESCC.

We found that the metabolic profiles of serum could differentiate patients with ESCC from CTRL using OPLS-DA methods. Altered metabolite levels could reflect disturbed glycometabolism (glycolysis and the TCA cycle), fatty acids, amino acids, choline, ketone bodies, nucleotides, and lipid metabolism. The potential biomarker panel, which reflects metabolic changes including glycolysis, choline, amino acids, and nucleotide metabolism, could significantly differentiate early ESCC patients from CTRL. One glucose molecule can generate 36 ATP molecules via the TCA cycle, while glycolysis produces only two (Fig. S2). The use of glucose by tumour cells to generate energy under conditions of adequate oxygen supply is called the Warburg effect (Warburg, 1956; Gillies, Robey & Gatenby, 2008). The significant decrease in glucose found in our study demonstrated that the metabolic feature of ESCC with strong aerobic glycolysis is consistent with the results observed in many other rapidly proliferating cancers (Theodorescu et al., 2006; Chen et al., 2011). Accelerated glycolysis is a characteristic of all types of cancer and altered glycolysis has been previously examined in ESCC. The metabolomic results showed that energy metabolism was the dominant factor in the pathophysiologic mechanism of ESCC. It also identified triglycerides, glycoproteins, and acetone as important sources of energy. Lactate levels were disregarded because of the chance of glycolysis occurring in serum samples during the experiment. Choline, with higher VIP values in patients with ESCC, was the second markedly altered metabolite. Choline, phosphorylcholine, and glycerophosphorylcholine (GPC) are important for the phospholipid metabolism of cell membranes and have been previously identified as markers of cell proliferation and growth. The increased choline and decreased GPC identified in our research were probably membrane breakdown products due to accelerated tumour propagation. This result is consistent with those obtained for other tumour types (Dobrzyńska et al., 2005; Monteggia et al., 2000; Chen et al., 2011), including those in the high-resolution magic-angle spinning ^1^H-NMR spectroscopy study of squamous carcinoma tissues (Yang et al., 2013). Elevated glutamine, glutamate, and glucogenic amino acid levels were also observed playing a distinct role in proliferating cancer cells in early stage ESCC. To provide for continuous high-energy demands for fast cell proliferation even under hypoxic conditions, glutamine is converted to glutamate, and further transformed into alpha-ketoglutarate for ATP synthesis through the TCA cycle (Zhang et al., 2013a; Zhang et al., 2013b), similarly shown in the results of other serum studies (Wu et al., 2009; Zhang et al., 2011; Ikeda et al., 2012; Wang et al., 2017; Zhang et al., 2017). However, our results are inconsistent with those previously reported for ESCC (Jin et al., 2014; Ma et al., 2014). One reason for this is that these studies focused on the signatures of lymph node ESCC metastasis in which glutamine/glutamate could be consumed in the TCA cycle. Another plausible reason for the observed discrepancies is that they used a different platform (UPLC/TOF/MS or GC/MS) and different status of tumor metabolism (Davis et al., 2012; Liu et al., 2013), whereas we only concentrated on the early stages of ESCC. A number of amino acid and amino acid derivatives consumed in the blood possibly represent cachexia-induced skeletal muscle protein breakdown in the early stages of ESCC. Compared with CTRL, we found that valine levels were significantly reduced in patients with ESCC. Valine is a branched-chain amino acid, which are essential amino acids that serve as nitrogen donors for nonessential amino acids and are important for energy consumption (Jin et al., 2014). The decreased valine levels in ESCC patients indicate the need for glutamine biosynthesis, related to the TCA cycle, in response to higher energy requirements for tumour proliferation. We also observed that DHT levels decreased more remarkably in early stage ESCC than in CTRL. DHT was identified by spiking because the discovery of this metabolite is one of the essential results of our study. DHT is an intermediate decomposition product of thymine (Pero, Johnson & Olsson, 1984). DNA replication in tumour cells rapidly exhausts thymine levels; hence, decreased DHT could be a feasible new biomarker associated with tumour occurrence (Hasim et al., 2012). Tumour burden can inhibit and lower the body’s immune function, and further promote the development of cancer. The results indicate that tumour burden causes the reprogramming of serum metabolites in ESCC patients. Understanding how these metabolite profiles differ should provide insight into early stage ESCC and identify new targets for treatment.

## Conclusions

The present study manifested the serum metabolic alterations of early stage of ESCC by NMR-based metabolomics method, which has convincingly contributed in both aiding diagnosis and affording new insights in regard to pathological mechanisms in ESCC. NMR has been proven to be a well-established, robust, and reproducible tool for non-invasive methods, and has provided valuable diagnostic information, and potential therapeutic targets for clinical therapeutics. However, it still has a relatively low sensitivity and a narrow dynamic range compared to other platforms (such as mass spectrometry). We did not achieve absolute quantification of metabolites in our experiment as the lipids and protein were not removed. In the future, using a combination of multiple metabolomic analysis platforms could give us a detailed picture of metabolic changes in ESCC patients compared with CTRL. Furthermore, understanding the molecular pathogenesis of serum metabolic alterations in ESCC could offer a new field for individualized cancer therapy and prognostic prediction. In the future, a large number of independent cohorts of patients with different varieties of cancer, along with CTRL, will be recruited to screen for potential biomarkers and to verify the findings reported here.

##  Supplemental Information

10.7717/peerj.8151/supp-1 Figure S1 The PLS-DA scores plots based on ^1^H CPMG NMR spectra of serum obtained from heathly controls (CTRL) and ESCC groups (*R*^2^*X* = 51.9%, *R*^2^*Y* = 80.9%, *Q*^2^ = 81.4%)

10.7717/peerj.8151/supp-2 Figure S2 The fragment described the oxophos and Warburg effect-anareobic glycolysis for early stage of ESCC Yellow arrows mean up-regulated with respect to CTRL, and blue arrows mean down-regulated with respect to CTRL.

10.7717/peerj.8151/supp-3 Supplemental Information 1 Raw data exported from NMR acquisition which applied for data analysis and preparation for Figs. 1 and 2, Fig. S1 and Table 2

## Additional Information and Declarations

### Data Availability

The following information was supplied regarding data availability: The NMR raw data are available in the Supplementary Files.

### Clinical Trial Registration

The following information was supplied regarding Clinical Trial registration: 2016-32
