# Supplementary figures and images for: 1H-NMR spectroscopy identifies potential biomarkers in serum metabolomic signatures for early stage esophageal squamous cell carcinoma
Source: PeerJ. 2019 Nov 29;7:e8151. doi: 10.7717/peerj.8151 (PMC6886491; doi:10.7717/peerj.8151)

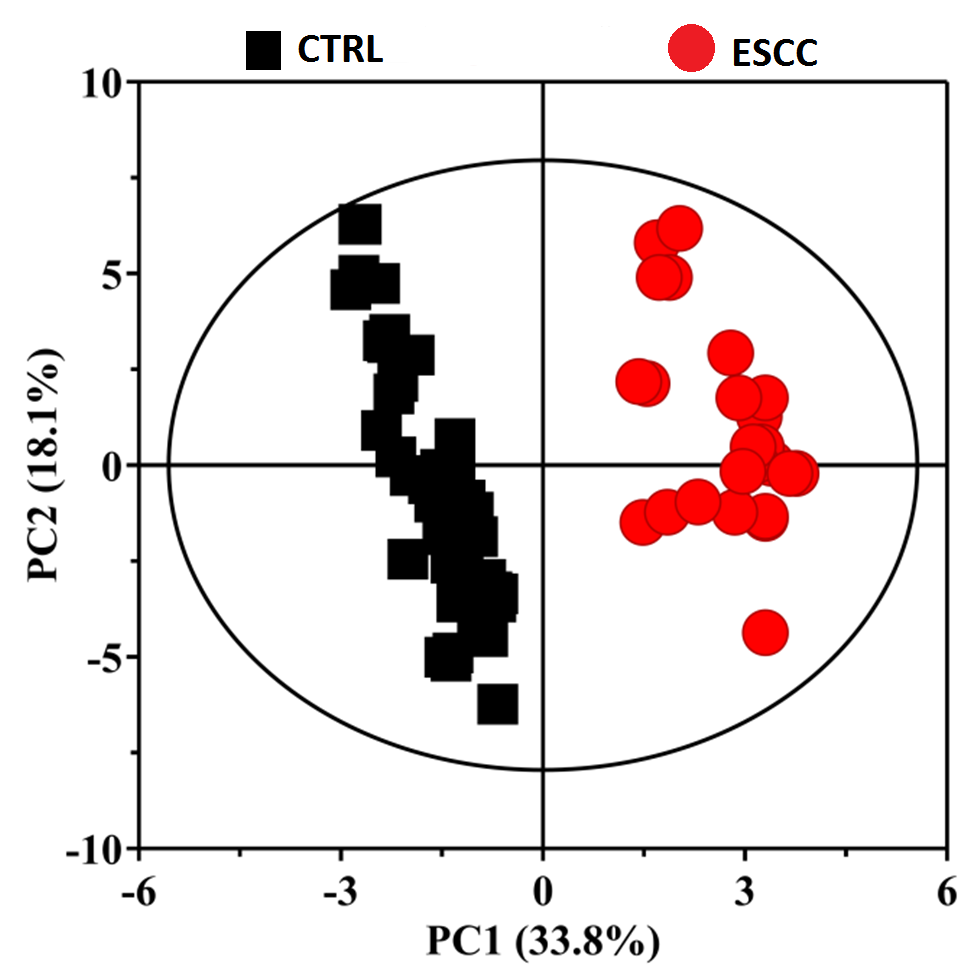

Supplement: Figure S1 [file peerj-07-8151-s001.png]

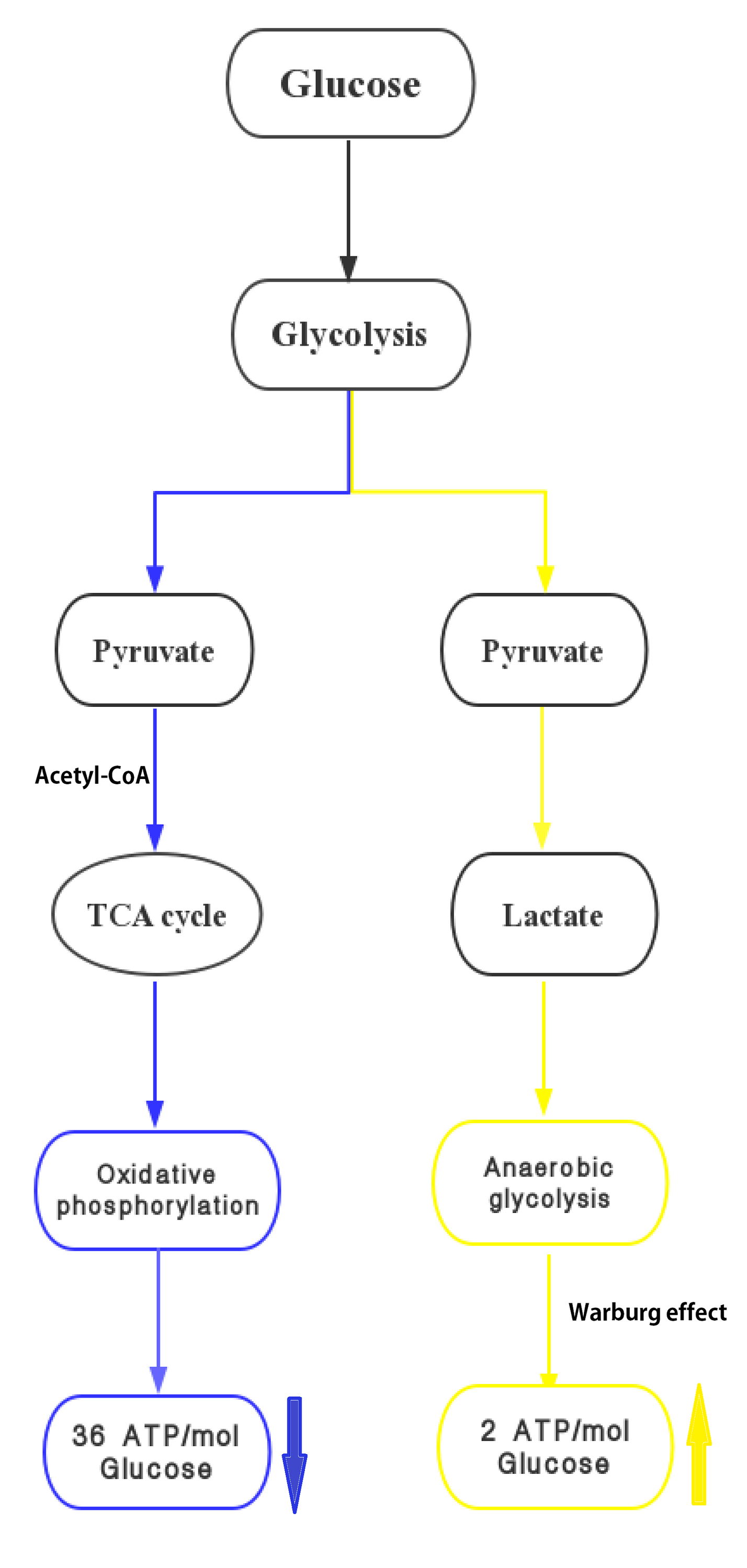

Supplement: Figure S2 — Yellow arrows mean up-regulated with respect to CTRL, and blue arrows mean down-regulated with respect to CTRL. [file peerj-07-8151-s002.png]
